# Enterotypical *Prevotella* and three novel bacterial biomarkers in preoperative stool predict the clinical outcome of colorectal cancer

**DOI:** 10.1186/s40168-022-01388-8

**Published:** 2022-11-28

**Authors:** Ji-Won Huh, Min Jung Kim, Jaesik Kim, Hyeon Gwon Lee, Seung-Bum Ryoo, Ja-Lok Ku, Seung-Yong Jeong, Kyu Joo Park, Dokyoon Kim, Jihyun F. Kim, Ji Won Park

**Affiliations:** 1 Department of Systems Biology, Division of Life Sciences, and Institute for Life Science and Biotechnology, Yonsei University, Seoul, Republic of Korea; 2 Division of Colorectal Surgery, Department of Surgery, Seoul National University College of Medicine, Seoul, Republic of Korea; 3 Cancer Research Institute, Seoul National University, Seoul, Republic of Korea; 4 Department of Computer Engineering, Ajou University, Suwon, Republic of Korea; 5 Department of Biostatistics, Epidemiology and Informatics, Perelman School of Medicine, University of Pennsylvania, Philadelphia, USA; 6 Department of Biomedical Sciences, Seoul National University College of Medicine, Seoul, Republic of Korea; 7 Institute for Biomedical Informatics, University of Pennsylvania, Philadelphia, USA; 8 Microbiome Initiative, Yonsei University, Seoul, Republic of Korea

**Keywords:** Gut microbiome, Metagenome, Microbial hazard score, Dysbiosis, QIIME

## Abstract

**Background:**

A significant proportion of colorectal cancer (CRC) patients suffer from early recurrence and progression after surgical treatment. Although the gut microbiota is considered as a key player in the initiation and progression of CRC, most prospective studies have been focused on a particular pathobionts such as *Fusobacterium nucleatum*. Here, we aimed to identify novel prognostic bacteria for CRC by examining the preoperative gut microbiota through 16S ribosomal RNA gene sequencing.

**Results:**

We collected stool samples from 333 patients with primary CRC within 2 weeks before surgery and followed up the patients for a median of 27.6 months for progression and 43.6 months for survival. The sequence and prognosis data were assessed using the log-rank test and multivariate Cox proportional hazard analysis. The gut microbiota was associated with the clinical outcomes of CRC patients (*P*_progress_ = 0.011, *P*_decease_ = 0.007). In particular, the high abundance of *Prevotella*, a representative genus of human enterotypes, indicated lower risks of CRC progression (*P* = 0.026) and decease (*P* = 0.0056), while the occurrence of *Alistipes* assigned to *Bacteroides* sp., *Pyramidobacter piscolens*, *Dialister invisus*, and *Fusobacterium nucleatum* indicated a high risk of progression. A microbiota-derived hazard score considering the five prognostic bacteria accurately predicted CRC progression in 1000 random subsamples; it outperformed widely accepted clinical biomarkers such as carcinoembryonic antigen and lymphatic invasion, after adjustment for the clinicopathological stage (adjusted HR 2.07 [95% CI, 1.61–2.64], *P* = 7.8e−9, C-index = 0.78). PICRUSt2 suggested that microbial pathways pertaining to thiamine salvage and L-histidine degradation underlie the different prognoses.

**Conclusions:**

The enterotypical genus *Prevotella* was demonstrated to be useful in improving CRC prognosis, and combined with the four pathobionts, our hazard score based on the gut microbiota should provide an important asset in predicting medical outcomes for CRC patients.

Video Abstract

**Supplementary Information:**

The online version contains supplementary material available at 10.1186/s40168-022-01388-8.

## Backgrounds

Colorectal cancer (CRC) is the third most common malignancy, and the second most common cause of cancer-related death worldwide; its incidence is steadily increasing in “Westernizing” countries [1]. Advances in screening and diagnostic techniques facilitate early detection. However, 24% of patients with stage I–III CRC relapse within 5 years after tumor removal, and more than half of the cases occur within 2 years [2]. Therefore, stratifying the post-surgical risk of progression is important for personalized and long-term management. The pathological stage of CRC is the most important factor in predicting the medical outcomes after curative resection; however, some modifiable factors measured from the blood and stool are useful indicators [3].

Trillions of microorganisms inhabit the gastrointestinal tract and influence the host physiology [4]. The disruption of host-microbial symbiosis is associated with disorders, such as chronic inflammation, metabolic syndromes, behavioral disorders, and cancers [5]. Gut microbiota is extensively implicated in the etiology of CRC [6]. *Fusobacterium nucleatum*, an anaerobic oral commensal, prevails in human colorectal carcinoma and promotes cancer progression in rodents [7, 8]. Pathogenic strains of *Escherichia coli* and *Bacteroides fragilis* are associated with host DNA damage and tumor-promoting chronic inflammation, respectively [9, 10]. Many case-control and cohort studies have been conducted to exploit gut microbes as prognostic biomarkers [11]; however, most of these studies used targeted PCR to detect specific pathobionts, mainly *F. nucleatum*. Several recent analyses using 16S ribosomal RNA (rRNA) gene sequencing searched for novel prognostic bacteria, which was not very successful [12–14].

To test if the gut microbiota harbors information on the state of patients with CRC, we performed the 16S rRNA gene-based community analysis of the gut microbiota of 333 CRC patients. Kaplan-Meier survival analysis suggested that *Prevotella*, a representative genus of human enterotypes [15], is significantly associated with a better prognosis of CRC. In addition to pre-documented *F. nucleatum*, three novel microbial indicators of poor prognosis were identified. The prognostic bacteria were combined to generate a microbial hazard score, which predicted CRC progression independently of other strong prognostic factors.

## Methods

### Patients and sample collection

We recruited 339 patients with colorectal neoplasms who were scheduled for elective surgical resection at Seoul National University Hospital. Fecal samples were collected within 2 weeks prior to surgery. Eligible patients were provided with a DNA/RNA ShieldTM fecal collection tube (Zymo Research Corp) to collect fecal samples at home. The fecal samples were sent to a laboratory within 24 h of collection. After the exclusion of six patients (two, carcinoma in situ; two, denial of surgery; and two, non-adenocarcinoma), 333 patients, who underwent primary tumor resection for colorectal cancer, were included in the analysis. All included patients had tumors confirmed as pathological adenocarcinoma. The clinical data of patients are provided in Table S1, which includes sex, age, body mass index (BMI), American Society of Anesthesiologists (ASA) classification, smoking history, alcohol consumption history, preoperative laboratory test results, neoadjuvant treatment, postoperative chemotherapy, medication, comorbidity, and detailed information of tumor—clinicopathological stage, TNM stages, lymphatic invasion, and mutational profiles (K-ras). Tumors located in the splenic flexure, descending colon, and sigmoid colon were defined as distal colon cancer, while those located in the cecum, ascending colon, hepatic flexure, and transverse colon were classified as proximal colon cancer.

Patients were monitored for cancer progression every 3 or 6 months. The median follow-up period was 29.5 months for 269 non-progressed patients (interquartile 23.8–31.8 months, 15 months for progressed cases). Physical examination and laboratory tests, including the level of serum carcinoembryonic antigen (CEA), were performed at each follow-up visit. Abdominopelvic and chest computed tomography was performed every 6 months or 1 year. Colonoscopy was performed every 1–2 years. Tumor recurrence or progression was identified through pathologic or radiologic examinations, or both. Overall survival was documented at the time of the submission state of this paper, and the median follow-up period was 43.6 months for survivors (interquartile 37.6–48.0 months, 25.6 months for deceased cases).

### Library construction and sequencing

Genomic DNA in feces was extracted using a QIAamp® Fast DNA Stool Mini Kit (Qiagen), according to the manufacturer’s instructions. Sequencing library protocols amplified variable regions 3 and 4 (V3-V4) of the 16S rRNA gene. The genomic DNA (2 ng) was PCR amplified using universal forward/reverse primers. The universal primer pair with Illumina adapter overhang sequences are as follows:

V3-F: 5′-TCGTCGGCAGCGTCAGATGTGTATAAGAGACAGCCTACGGGNGGCWGCAG-3′

V4-R: 5′-GTCTCGTGGGCTCGGAGATGTGTATAAGAGACAGGACTACHVGGGTATCTAATCC-3′

The products from the first-round PCR amplification were purified using AMPure beads (Agencourt Bioscience), and 2 μl of the purified product was amplified using the NexteraXT Indexed Primer. The purified final product was quantified using the qPCR Quantification Protocol Guide and analyzed using the TapeStation D1000 ScreenTape (Agilent Technologies). Paired-end (2×300 bp) sequencing was performed using the Illumina MiSeq™ platform.

### 16S rRNA gene sequencing analysis

A total of 34,184,627 read counts for 17,194 amplicon sequence variants (ASVs) were retrieved using the QIIME2 pipeline [16]. The abundance table of ASVs was constructed using the DADA2 denoising algorithm after trimming off the sequencing primer sequences [17]. For the taxonomic assignment, reference sequences and taxonomy of 99% operative taxonomic units were obtained from the latest SILVA 138 database and were trained towards the V3-V4 amplicon [18]. The abundance of ASVs was rarefied to make the library size even by using the rarefy function in the vegan R package. The sampling size was the minimum read count across the samples (64,604). Alpha diversities, such as the observed number of ASVs and Shannon diversity index, were computed using the diversity function of the vegan R package.

To visualize beta diversity, Bray-Curtis distances between samples were calculated and displayed on the ordination plots of principal coordinates analysis (PCoA). Permutational multivariate analysis of variance (PERMANOVA) was performed using the adonis2 function to test whether the samples were differently dispersed by the indicated variables. A total of 333 samples were grouped into two clusters based on beta diversity using k-means clustering. The number of clusters (*k* = 2) was determined heuristically.

### Survival analysis

Progression-free survival (PFS) was defined as the time from surgery to any recurrence or progression of colorectal cancer or death from any cause. Survminer and survival R packages were used for Kaplan-Meier survival analysis. The log-rank test was used to compare survival by categorical variables, and continuous variables such as levels of serum metabolites, number of tissue-associated T cells, and bacterial counts were dichotomized at the median value. However, some variables followed previously suggested criteria: CEA, high > 5 ng/ml; plasma fibrinogen, high ≥ 335 mg/dL; neutrophil-to-lymphocyte ratio (NLR), high ≥ 5; platelet-to-lymphocyte ratio, high ≥ 150; lymphocyte-to-monocyte ratio, high ≥ 2.4; prognostic nutritional index, high ≥ 45; age, high ≥ 65; and BMI, high ≥ 25. Cox proportional hazard regression models (CoxPH) were generated to quantify the hazard ratio (HR) of host variables using the coxph function in the survival R package. Continuous variables were scaled before being applied to the model.

The reporting recommendations for tumor marker prognostic studies (REMARK) criteria were considered in this study [19]. In terms of sample size for PFS analysis, when the number of progression events (64) was compared with the number of a prognostic factor applied to multivariate CoxPH, “a minimum of 10 events per predictor” rule was satisfied [20].

### Screening prognostic bacteria

Among the 438 bacterial species observed, 79 species that appeared in 10% of the total samples with relative abundance higher than 0.01% were selected for screening prognostic pathobionts. The relative bacterial abundance was dichotomized at the median, which was applied to the univariate survival log-rank survival test and to multivariate CoxPH. For a robust screening, random subsets of the whole data (1000 subsampling) were used as well as the entire data itself. In the Monte Carlo cross-validation (CV), prognostic species with a *P* value lower than 0.05 in a discovery set (70%, 233/333) were confirmed in a validation set (30%); the number of consistent discrimination in both discovery and validation sets was used to measure the robustness of each species. To remove confounding effects, Cox models were adjusted for TNM stages (T stage, 0–2 vs. 3/4; N stage, 0 vs. 1/2; M stage, 0 vs. 1), neoadjuvant treatment, postoperative chemotherapy, lymphatic invasion, and the binarized level of CEA. The *P* values from the log-rank test were adjusted using the Benjamini-Hochberg procedure. Four species that show a high effect size (HR > 1.5) and significance (FDR < 0.1) were selected as a prognostic biomarker.

### Microbiota-based hazard score

To generate risk-evaluating scores in a microbiota-focused manner, five bacteria were selected: *Prevotella* genus, *Bacteroides* sp., *P. piscolens*, *D. invisus*, and *F. nucleatum*. In the case of *Prevotella*, a score of 1 was assigned to the samples with a low abundance of *Prevotella* (relative abundance < 16.1%), and a score of 0 was assigned to the samples with high *Prevotella*. If a sample contained *Bacteroides* sp., *P. piscolens*, *D. invisus*, and *F. nucleatum*, a score of 1 was assigned to each bacterial species. By adding up the scores, 31 different hazard scores were generated. To compare the performance of the scores, half of the samples (166 out of 333) were randomly sampled 1000 times without replacement to form a pool of test datasets. The HR and log-rank significance of each score were calculated for the test datasets, and the variance of HRs across the test datasets was used as an indicator of model stability. For clear stratification, a six-layered hazard score (M5), among the best performers, was categorized by assigning scores 1 and 2 into the M5-moderate group and scores 3 to 5 into the M5-high group.

For comparison of prognostic indicators, pre-documented prognostic indicators and microbiota-derived biomarkers were added to a baseline hazard model constructed with the clinicopathological stage of CRC (stage 0–2 vs. stage 3/4) and tested whether the added variable improved the model in terms of concordance index. Numerical variables such as M5, CEA, age, fibrinogen, *Prevotella*, BMI, and NLR were also tested after conversion into categorical variables.

### Metagenomic functionality inference

Phylogenetic Investigation of Communities by Reconstruction of Unobserved States (PICRUSt2) was performed to infer metagenomic capacity [21]. To estimate the effect size, the average taxonomic relative functional abundance (TRFA) of each pathway across M5-moderate and M5-high samples was subtracted from the average TRFA in M5-low samples. A total of 15 microbial pathways (nine enriched and six depleted in M5-low) with false discovery rate (FDR) < 0.001 and absolute difference in average TRFA > 5 were selected as differentially abundant pathways. Pathway information was obtained from the MetaCyc database [22].

### Statistics

PERMANOVA was used to measure the association between clinical parameters and the gut microbiota based on the microbial Bray-Curtis dissimilarity. The Wilcoxon rank-sum test was used to compare the levels of continuous variables by categorical variables such as enterotype, cancer stage, progression, and M5-high versus M5-low. The Fisher’s exact test was used for measuring the association between two categorical variables; for those not suitable for the Fisher’s exact test, the chi-square test was used instead. Missing data in certain variables was removed. All steps for statistical analysis and visualization were performed in R studio (version 4.1.0) using indicated packages.

## Results

### Preoperative gut microbiota of CRC patients is associated with the state of the patients

**Fig. 1:**
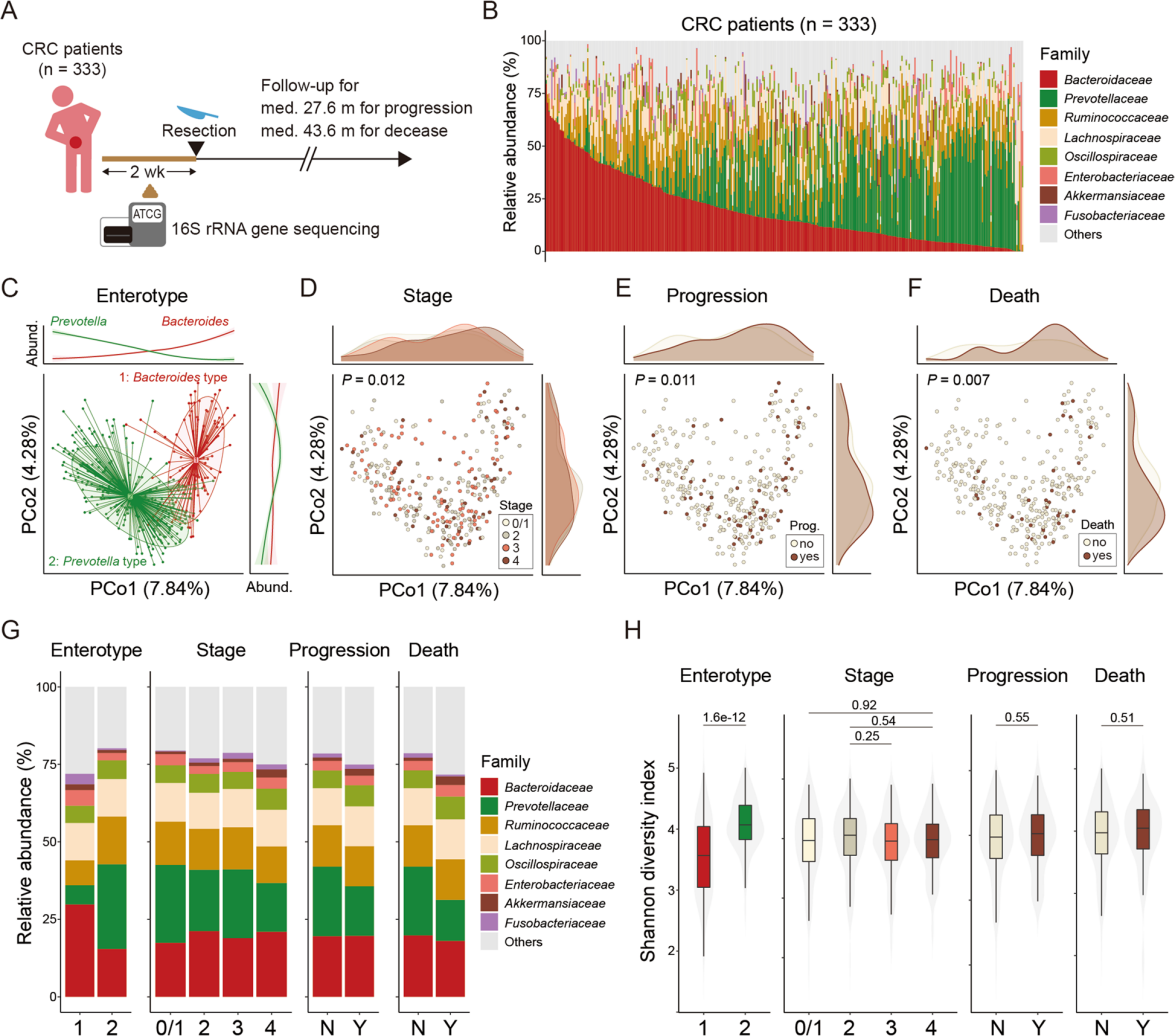
Preoperative gut microbiota of colorectal cancer patients. **A** Illustration of the study. **B** Taxonomic profile at the family level. Samples are arranged in the descending order of relative abundance of *Bacteroidaceae* family. **C–F** PCoA plots of the gut microbiota based on the Bray-Curtis dissimilarity. Samples were colored based on their enterotype (**C**), clinicopathological stage (**D**), progression (**E**), and decease (**F**), respectively. PERMANOVA was performed for *P* value calculation. **G** Taxonomic profile at the family level by indicated variables. **H** Shannon diversity index by indicated variables

To understand the prospective association between colorectal cancer (CRC) and gut microbiota, we recruited 333 patients with primary CRC who were scheduled for surgical removal of the cancer and obtained fecal samples from them within 2 weeks besfore the surgery (Fig. 1A). The clinical data of the patients are described in Table 1 and additional Table S1.

**Table 1:** Clinical parameters of CRC patients

	Total (***N*** = 333)
**Age (years, range)**	63 (28–87)
**Sex (male/female)**	209/124
**BMI (kg/m**^**2**^**, mean ± SD)**	24.3 ± 3.2
**Smoking (*n*, %)**	47 (14.1%)
Was	18 (5.4%)
**Drinking (*n*, %)**	121 (36.3%)
Was	4 (1.2%)
**ASA classification**	
1	110 (33.0%)
2	198 (59.5%)
3	25 (7.5%)
**Comorbidity (*n*, %)**	175 (52.6%)
Hypertension	126 (37.8%)
Type 2 diabetes	69 (20.7%)
Pulmonary disease	23 (6.9%)
**Medication (*n*, %)**	182 (54.7%)
anti-hypertensive	126 (37.8%)
anti-diabetic	58 (17.4%)
anti-inflammatory (nonsteroidal)	42 (12.6%)
**CEA-high (*n*, %)**	65 (19.9%)
**Tumor location**	
Proximal colon	68 (20.4%)
Distal colon	166 (49.6%)
Rectum	95 (28.5%)
**Tumor stage**	
0,1	76 (22.8%)
2	102 (30.6%)
3	108 (32.4%)
4 (M1)	47 (14.1%)
**Lymphatic invasion (*n*, %)**	98 (29.4%)
**K-ras mutation (*n*, %)**	101 (33.9%)
**MSI (*n*, %)**	40 (13.0%)
MSS/MSI-L/MSI-H	267/26/14

*MSI* microsatellite instability (*N* = 307)

Similar to healthy humans [15], the gut microbiota of CRC patients was clustered into *Bacteroides*-dominant enterotype 1 and *Prevotella*-dominant enterotype 2 with a reciprocal distribution of the representative genera (Fig. 1B, C). In PCoA, microbial diversity between the samples was associated with the clinicopathological stage (*P* = 0.012), the progression of CRC (*P* = 0.011), and the decease (*P* = 0.007; Fig. 1D–F). In addition, TNM staging systems of CRC were associated with the gut microbiome (*P*_T-stage_ = 0.012, *P*_N-stage_ = 0.063, *P*_M-stage_ = 0.01). The bacterial families of *Akkermansiaceae* and *Fusobacteriaceae* tended to increase in the patients with progression (*P*_*Akkermansiaceae*_ = 0.0039, *P*_*Fusobacteriaceae*_ = 0.1575), where *Prevotellaceae* decreased (*P* = 0.0228; Fig. 1G). The Shannon’s diversity index was significantly higher in enterotype 2; however, it was comparable between the CRC stages or postoperative outcomes (Fig. 1H). The proportion of patients with advanced CRC or with progression was higher in enterotype 1 with quasi-significance; the influence of sex was statistically irrelevant (*P*_stage_ = 0.0896, *P*_progress_ = 0.0476, *P*_decease_ = 0.1311, *P*_sex_ = 0.6173; Figure S1A–D). Among 35 blood variables measured, the levels of CEA and fibrinogen were significantly elevated in enterotype 1 (*P*_CEA_ = 0.0085, *P*_fibrinogen_ = 0.0022; Figure S1E–I). In contrast, several parameters such as neoadjuvant treatment, postoperative chemotherapy (193 adjuvant and 40 palliatives), the responses to the palliative chemotherapy (27 responders), comorbidity, and use of any medication at the time of stool sampling were not associated with the gut microbiome (*P*_neoadjTx_ = 0.728, *P*_post-chem_ = 0.11, *P*_response_ = 0.205, *P*_comorbidity_ = 0.192, *P*_medication_ = 0.149). Among the seven types of common drugs that were administered to at least 20 CRC patients, drugs for diabetes and peptic ulcer showed significant association with the microbiome (*P*_anti-diabetes_= 0.01, *P*_anti-ulcer_ = 0.025), whereas others—namely, anti-hypertensive drug, lipid-lowering drug, nonsteroidal anti-inflammatory drug, anti-platelet drug, and drug for benign prostatic hypertrophy—were not (data not shown).

### Enterotypical Prevotella and four opportunists indicate a state of CRC patients

**Fig. 2:**
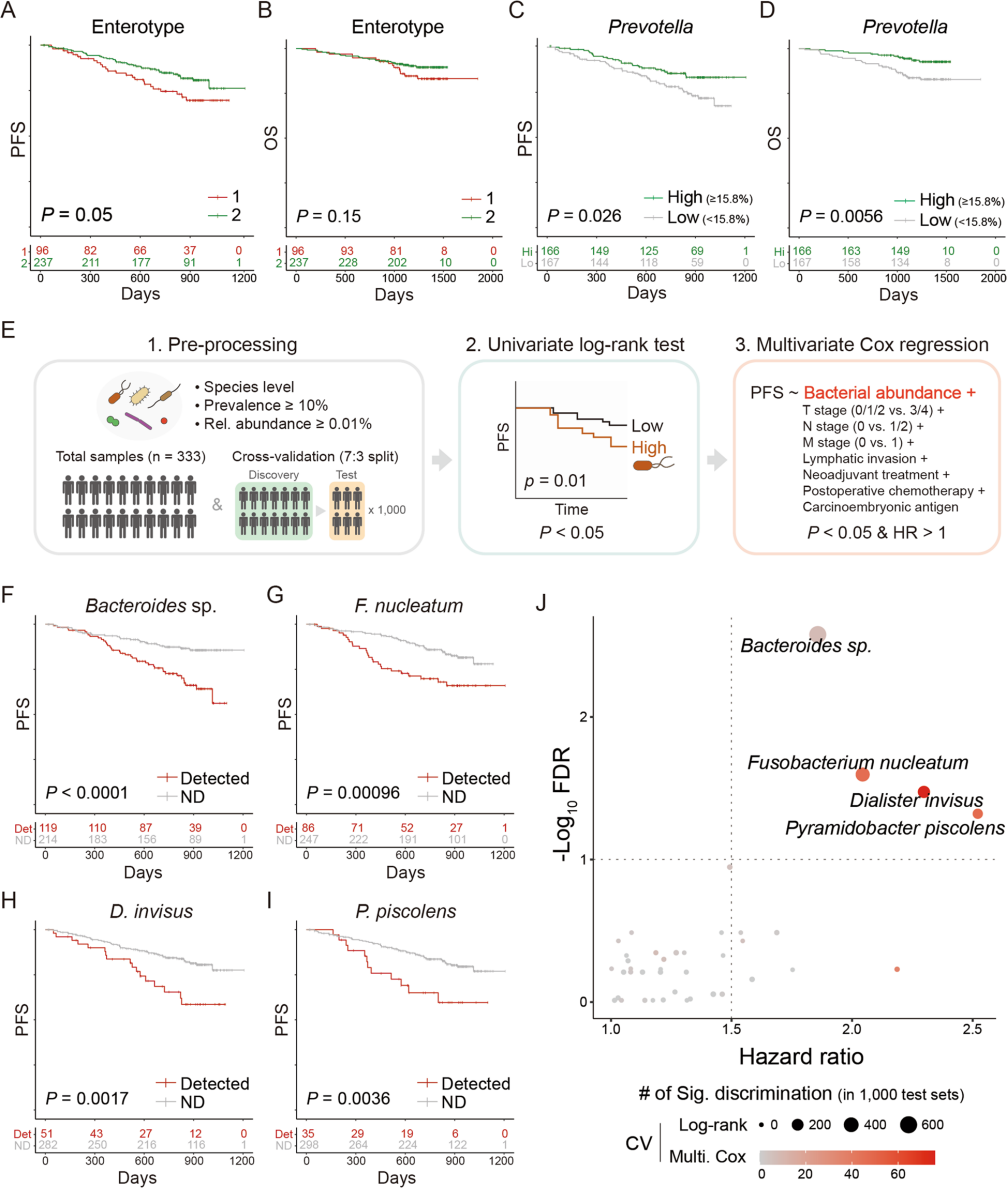
Screening prognostic microbial biomarkers. **A** PFS curve by enterotype. Integers 1 and 2 indicate *Bacteroides*-enriched enterotype 1 and *Prevotella*-enriched enterotype 2, respectively. **B** OS curve by enterotype. **C** PFS curve by the dichotomized relative abundance of *Prevotella*. **D** OS curve by the dichotomized relative abundance of *Prevotella*. **E** Schematic description for the screening of prognostic bacterial species. Dichotomized bacterial abundance is applied to both univariate log-rank test and multivariate Cox proportional hazard model. The entire data and random subsamples were used for biomarker screening. **F–I** PFS curves by the presence of *Bacteroides* sp. (**F**), *Fusobacterium nucleatum* (**G**), *Dialister invisus* (**H**), and *Pyramidobacter piscolens* (**I**). ND indicates not detected. The numbers at risk are presented under the survival curves. **J** HR of candidate prognostic species. Color and size indicate the number of significant discrimination in both discovery and test sets by univariate log-rank test and multivariate Cox model, respectively

The patients were followed up for the median of 27.6 months for CRC progression and the median of 42.7 months for survival. Then, we tested whether the enterotype and its representative bacteria were associated with CRC prognosis. Enterotype 1 showed shorter progression-free survival (PFS) and overall survival (OS) than enterotype 2 (*P*_PFS_ = 0.05, *P*_OS_ = 0.15; Fig. 2A, B). Among the enterotypical bacteria, the patients with a higher abundance of *Prevotella* had a lower risk of CRC progression and death (*P*_PFS_ = 0.026, *P*_OS_ = 0.0056; Fig. 2C and D); however, the abundances of *Bacteroides* (*P*_PFS_ = 0.39, *P*_OS_ = 0.45) and *Faecalibacterium* (*P*_PFS_ = 0.95, *P*_OS_ = 0.77) had little impact on PFS and OS (Figure S2A and B). Patients with CRC have microbial dysbiosis with an increased appearance of opportunistic pathobionts [23]. Therefore, we hypothesized that patients with poor prognosis harbor more pathobionts. We performed a univariate log-rank survival test and multivariate Cox proportional hazard analysis (CoxPH) for 79 species with a prevalence ≥ 10% and relative abundance ≥ 0.01%. The screening was conducted using both total samples and random subsamples (1000 times) (Fig. 2E). Log-rank test with total samples revealed that *Bacteroides* sp., *F. nucleatum*, *Dialister invisus*, and *Pyramidobacter piscolens* indicate an increased risk of progression and death (Fig. 2F–J and Figure S2C); those species significantly predicted disease progression after adjustment for multiple confounding factors—TNM stages, lymphatic invasion, neoadjuvant treatment, postoperative chemotherapy, and the level of CEA (adjusted HR_*P.pis*_ 2.52 [aHR_*P.pis*_], aHR_*D.inv*_ 2.30, aHR_*F.nuc*_ 2.04, HR_*B.*sp._ 1.86; Fig. 2J). In the random subsampling method, also known as the Monte Carlo CV, the number of consistent discrimination (*P* < 0.05) in both discovery and validation sets was used to measure the robustness of biomarker species. As a result, the four prognostic biomarkers were the most overrepresented ones in log-rank test and multivariate CoxPH (Fig. 2J), which collectively suggested that they are potent and robust biomarkers.

Among the eight ASVs assigned to *Bacteroides* sp. by the SILVA 138 database, seven ASVs belonging to the genus *Alistipes* covered all patients who are positive of *Bacteroides* sp. (Figure S3A). A phylogenetic tree was generated with the 16S rRNA gene amplicon sequences of *Bacteroides* sp. and 14 type strains of *Alistipes* species. The *Alistipes* ASVs assigned to *Bacteroides* sp. consisted of *A. dispar* and *A. senegalensis* (Figure S3C). Both the species distinguished poor outcomes as separate indicators (*P*_*A. dispar*_ = 0.00012, *P*_*A. senegalensis*_ = 0.017; Figure S3D and E).

### A gut microbiota-derived hazard score accurately predicts CRC prognosis

**Fig. 3:**
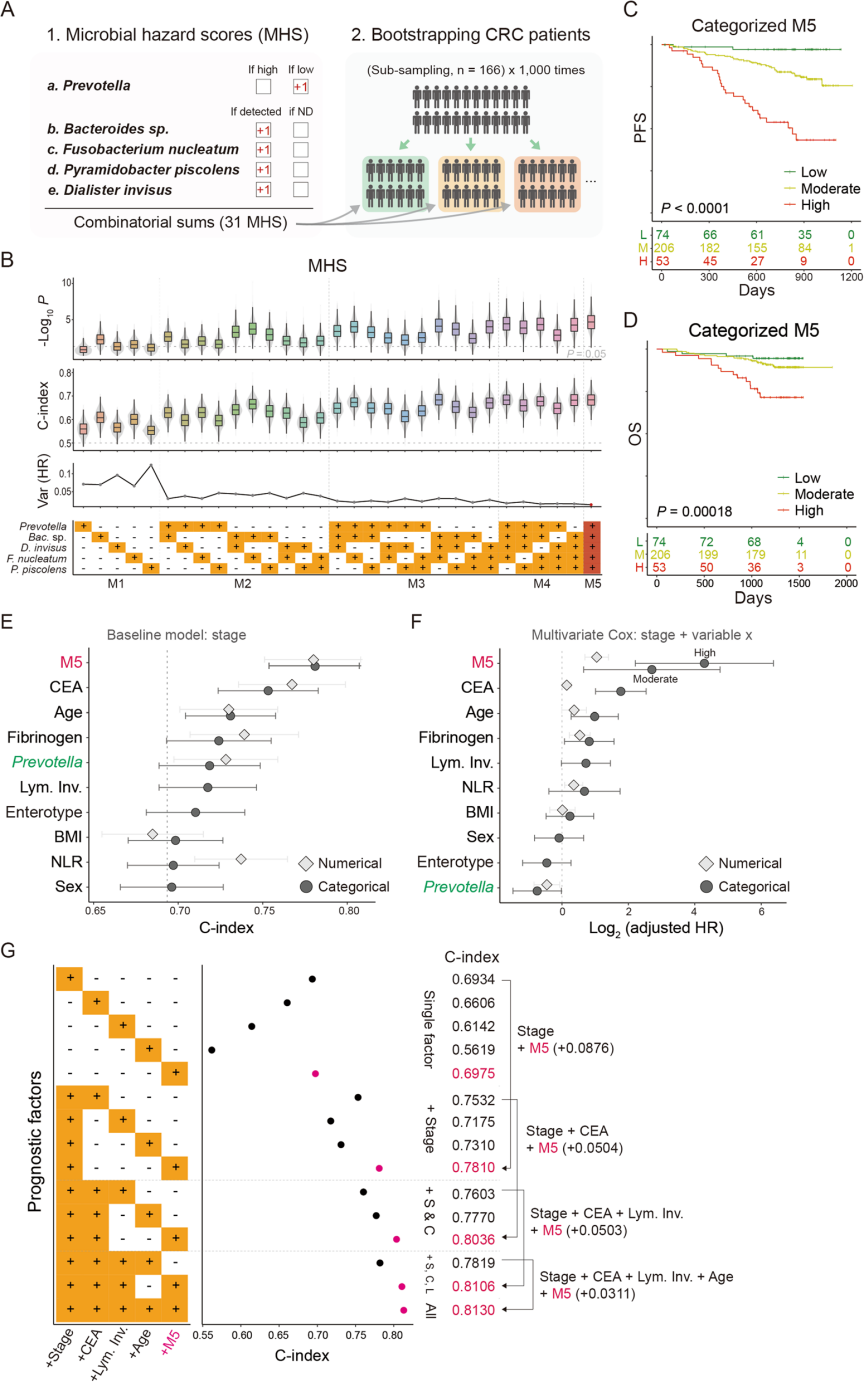
Performance of microbiota-derived hazard score. **A** Schematic illustration for the generation of microbial hazard scores (MHS). **B** Performance comparison of 31 MHS in bootstrapped datasets. Logarithmic *P* values for the log-rank survival test (top), C-index (middle), and variance of logarithmic HR (bottom) are displayed. **C** PFS curve by categorized M5. **D** OS curve by categorized M5. **E** Performance comparison of M5 with other indicators. The baseline model was constructed using the cancer stage; C-index is indicated as the dotted line. Other categorized variables indicate the HR of the high-group over low group. **F** Adjusted HRs of the indicated biomarkers. Variables were adjusted by the pathological stage of cancer. Enterotype showed the HR of *Prevotella* type 2 over *Bacteroides* type 1. **G** C-index of combinatorial prognostic factors. Parenthesis includes the increased C-index following the addition of M5 to the previous model. Lym. Inv.: lymphatic invasion. The numbers at risk are presented under the survival curves

To generate a robust microbial hazard score (MHS) by combining the prognostic bacteria, we randomly sampled half of the original dataset 1000 times; we applied 31 different combinations of MHS to the pool of test datasets (Fig. 3A). Among the scores, M5, considering the abundance of *Prevotella* and occurrence of the four pathobionts, showed the highest significance and concordance index (C-index) and the smallest variance of HR (median *P*_M5_ = 2.1e−05, C-index 0.68 ± 0.03, mean HR 2.04, minimum HR 1.41, variance of HR 1.01; Fig. 3B). The M5 stratified patients in a stepwise manner based on their risk (Figure S2D and E). For better separation, we regrouped M5 scores into three categories: 0 for M5-low, 1 and 2 for M5-moderate, and scores 3 to 5 for M5-high, which clearly distinguished the high- and low-risk groups of patients (Fig. 3C, D). The M5-low patients, who carry a high abundance of *Prevotella* and no pathobionts, had a low rate of progression with only two progressed cases out of 74. Furthermore, the M5-high group had undesirable manifestations compared to the M5-low: old age, systemic illness, concurrent disorders, advanced cancer stage, lymphatic invasion, and K-ras mutation (Table 2). Especially, type 2 diabetes showed increasing concurrence with higher M5: 13.5% in M5-low, 21.4% in M5-moderate, and 28.3% in M5-high (Table 2). These data may imply the convergent characteristics between the gut microbiotas of CRC and diabetes.

**Table 2:** Clinical parameters of CRC patients by M5 category

M5 category	Low (***n*** = 74)	Moderate (***n*** = 206)	High (***n*** = 53)	***P*** value
**Age (years, range)**	61 (38–81)	62 (28–87)	68 (40–83)	0.0656
**Sex (male/female)**	23/51	79/127	22/31	0.4324
**BMI (kg/m**^**2**^**)**	24.63 ± 2.79	24.33 ± 3.28	24.03 ± 3.33	0.568
**Smoking (*n*, %)**	10 (13.5%)	32 (15.5%)	5 (9.4%)	0.08367
**Drinking (*n*, %)**	35 (47.3%)	78 (37.9%)	8 (15.1%)	0.001077
**ASA classification**				
1	27 (36.5%)	70 (34.0%)	13 (24.5%)	0.1786^&^
2	44 (59.5%)	122 (59.2%)	32 (60.4%)	
3	3 (4.1%)	14 (6.8%)	8 (15.1%)	
**CEA-high (*n*, %)**	9 (12.2%)	34 (16.9%)	30 (57.7%)	0.0001248
**Comorbidity (*n*, %)**	42 (56.8%)	99 (48.1%)	34 (64.2%)	0.07785
Hypertension	36 (48.6%)	68 (33%)	22 (41%)	0.04865
Type 2 diabetes	10 (13.5%)	44 (21.4%)	15 (28.3%)	0.119
Pulmonary disease	6 (8.1%)	15 (7.3%)	2 (3.8%)	0.644
**Medication (*n*, %)**	43 (58.1%)	104 (50.5%)	35 (66.0%)	0.1042
Anti-hypertensive	31 (42.9%)	71 (34.5%)	24 (45.3%)	0.235
Anti-diabetic	7 (9.5%)	39 (18.9%)	12 (22.6%)	0.08794
Anti-inflammatory	7 (9.5%)	24 (11.7%)	11 (20.8%)	0.1477
**Tumor location**				
Proximal colon	22 (29.7%)	38 (18.4%)	8 (15.1%)	0.09491^&^
Distal colon	29 (39.2%)	107 (51.9%)	30 (57.7%)	
Rectum	22 (29.7%)	60 (29.1%)	13 (24.5%)	
**Tumor stage**				
0,1	23 (31.1%)	50 (24.3%)	3 (5.7%)	0.002322^&^
2	19 (26.8%)	67 (32.5%)	16 (30.2%)	
3	26 (35.1%)	63 (30.6%)	19 (35.8%)	
4 (M1)	6 (8.1%)	26 (12.6%)	15 (28.3%)	
**Lym. Inv. (*n*, %)**	18 (24.3%)	62 (30.1%)	18 (34.0%)	0.4769
**MSI (*n*/*N*, %)**	14/66 (21.2%)	23/188 (12.2%)	3/53 (5.7%)	0.00797
**K-ras (*n*, %)**	22 (33.3%)	60 (33.0%)	19 (38.0%)	0.8157

The anti-inflammatory drug is nonsteroidal. One-way ANOVA is used for continuous variables. Fisher’s exact test is a default test for comparing categorical variables unless specified otherwise. $ indicates that the variable is unsuitable for Fisher’s exact test and applied to chi-square test. *Lym. Inv.*, lymphatic invasion; *MSI*, microsatellite instability

We confirmed the prognostic value of several pre-documented biomarkers including age [24], clinicopathological stage [25], lymphatic invasion [26], levels of circulating CEA [27], fibrinogen [28], bilirubin [29], and CRP [30] (Figure S4A); however, blood inflammation-derived biomarkers such as NLR [31], lymphocyte-to-monocyte ratio [32], prognostic nutrition index [33], and platelet-to-lymphocyte ratio [34] did not correlate with the cancer progression (Figure S4B).

To compare the performance of M5 with the pre-documented biomarkers, we tested the extent to which an additional indicator improved the baseline model constructed on the cancer stage. Ten biomarkers were tested: CEA, fibrinogen, age, lymphatic invasion, BMI, NLR, sex, and three microbiota-related indicators (M5, enterotype, and the dichotomized abundance of *Prevotella*). M5 outperformed the other indicators regardless of the variable type (C-index_base_ = 0.69, C-index_+M5_ = 0.78, aHR_M5-high_ 19.54, aHR_M5-moderate_ 6.53, aHR_M5-num_ 2.07; Fig. 3E, F). In addition to CEA and age, the abundance of *Prevotella* improved the baseline model (C-index_+*Prevotella*_ = 0.73; Fig. 3E). We added more indicators to check the redundancy; M5 independently enhanced the power of prediction with extra indicators (Fig. 3G). The increase in C-index by M5 was comparable with the concerted consideration of CEA, lymphatic invasion, and age (ΔC-index_Stage+M5_ +0.0876, ΔC-index_stage+CEA/Lym. Inv./Age_ +0.0885; Fig. 3G), which supports strong efficacy of the M5.

### Microbial metabolism correlates with different prognoses

**Fig. 4:**
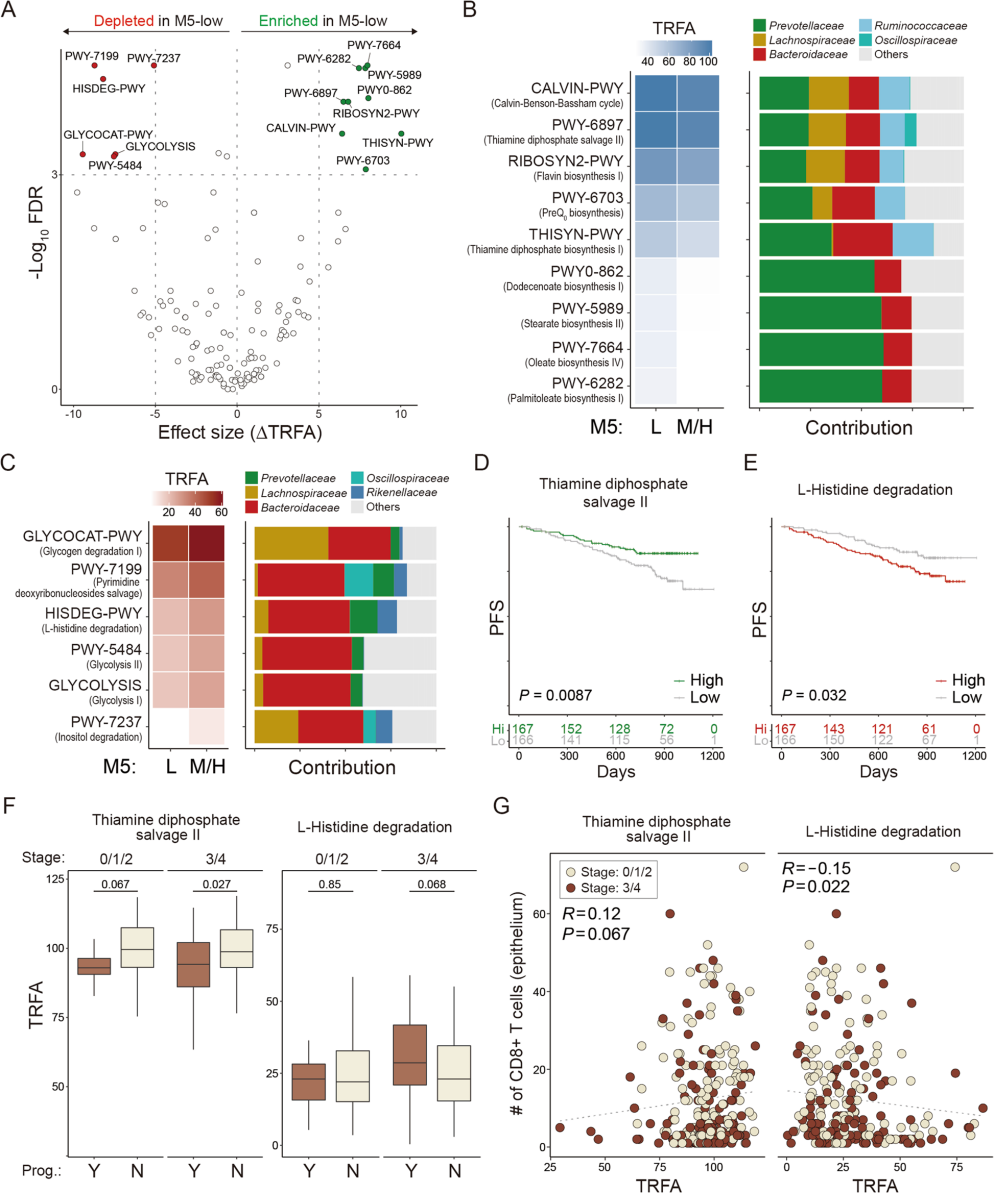
Metagenome inference of microbial pathways. **A** Screening prognosis-associated metagenome pathways using PICRUSt2. Wilcoxon test was performed to examine the distribution of microbial pathways. Differential TRFA was regarded as the effect size. **B** TRFA and taxonomic contribution of pathways enriched in the M5-low patients. **C** TRFA and taxonomic contribution of pathways depleted in the M5-low patients. **D, E** PFS curves by thiamine diphosphate salvage II pathway (PWY-6897) (**D**) and L-histidine degradation pathway (HISDEG-PWY) (**E**). **F** TRFA of the two significant microbial pathways by clinical stage and recurrence of CRC. **G** Spearman’s correlation between TRFA and the number of CD8^+^ T cells in the epithelium and its significance. The numbers at risk are presented under the survival curves

To define microbial metabolic pathways underlying the different outcomes, the metagenomic capacity was inferred using PICRUSt2. The enrichment and depletion of the inferred pathways in the M5-low group were tested. The difference in taxonomic relative functional abundance (TRFA) between the M5-low and M5-moderate/high groups was used as the effect size of each pathway. Nine beneficial and six detrimental pathways were suggested (FDR < 0.001, ΔTRFA > 5; Fig. 4A). The beneficial pathways included Calvin-Benson-Bassham cycle, thiamine diphosphate salvage II, thiamine diphosphate biosynthesis I, flavin biosynthesis I, preQ_0_ biosynthesis, dodecenoate biosynthesis I, stearate biosynthesis II, oleate biosynthesis IV, and palmitoleate biosynthesis I. The detrimental pathways included glycogen degradation I, glycolysis I, glycolysis II, pyrimidine deoxyribonucleoside salvage, L-histidine degradation I, and myo-, chiro-, and scyllo-inositol degradation (Fig. 4B, C). Among them, two pathways—thiamine diphosphate salvage II (*P* = 0.0087) and L-histidine degradation I (*P* = 0.032)—significantly discriminated among the outcomes (Fig. 4D, E). The taxonomic contribution of thiamine diphosphate salvage II was attributable to various bacterial families, which indicates the microbial consortium that forms a favorable niche for CRC patients cooperatively (Fig. 4B). In contrast, the detrimental pathways were mainly derived from the *Bacteroidaceae* family (Fig. 4C). The TRFA of the thiamine diphosphate salvage II pathway was lower in progressed patients regardless of cancer stage; however, the TRFA of L-histidine degradation was elevated only in the progressed patients at the later stage (Fig. 4F). Notably, both pathways correlated with the number of cytotoxic T cells in tumor-associated epithelium, implying their immunomodulatory potential (Spearman’s ρ_thiamine_ = 0.12, *P*_thiamine_ = 0.067, ρ_L-histidine_ = −0.15, *P*_L-histidine_ = 0.022; Fig. 4G). Among the remaining pathways, flavin biosynthesis I (*P* = 0.05), preQ_0_ biosynthesis (*P* = 0.06), Calvin cycle (*P* = 0.082), and thiamine diphosphate biosynthesis I (*P* = 0.087) were related to improved prognosis, whereas the two glycolysis pathways (*P*_glycolysisI_ = 0.051, *P*_glycolysisII_ = 0.085) were negatively correlated with marginal significance (Figure S5). A careful approach, however, is necessary to interpret the results because the taxonomic composition not always reflects the true functional capacity of the gut microbiota, while these data may provide clues to search for potential postbiotics for CRC patients.

## Discussion

We analyzed the gut microbiota of CRC patients and identified novel prognostic bacteria, including *Prevotella*, two *Alistipes* spp., *P. piscolens*, and *D. invisus*, in addition to the already implicated *F. nucleatum*. Expanding the repertoire of non-invasive biomarkers is necessary for early diagnosis and proper prognosis of CRC; however, only a few studies have profiled the overall microbiota through 16S rRNA gene sequencing [11]. The prognostic value of *F. nucleatum* and *B. fragilis* was reproduced in a study sequencing 180 CRC biopsies [12]; slightly improved survival was observed in 23 patients with the high *Prevotella* co-abundance group, albeit insignificant [13]. We show for the first time the significant association between an enterotypical genus *Prevotella* and CRC prognosis.

Human gut microbiota is classified into two enterotypes, represented by the genera *Bacteroides* and *Prevotella* [35]. Although the enterotype is highly affected by the host lifestyle, it can be altered through appropriate intervention [36]. The *Prevotella* is enriched in the gut of non-Western people, who consume more vegetables and less processed meat [37]. Despite the generally accepted benefits of the non-Western diet in preventing CRC, the relationship between the plant-favoring *Prevotella* and CRC has been debated [38–40]. This disparity could be attributed to the demographic differences in the genetic and environmental factors or to the intra-genus heterogeneity across populations. A recent cohort study in the USA revealed that higher fiber intake after CRC diagnosis reduced mortality, implying a beneficial effect of *Prevotella* on CRC prognosis [41]. In our Korean cohort, *Prevotella* was detected in 90.7% of the patients with CRC (302/333) with a median abundance of 15.8%; however, for global application of the *Prevotella*-considering M5, the dichotomization criterion needs further confirmation in a Western dataset.

The oral microbiota has emerged as a predictive biomarker for CRC and as an important source of pathogens for gastrointestinal disorders [42, 43]. Indeed, three of our detrimental biomarkers were included in the oral microbiota. *F. nucleatum* and *D. invisus* are readily found in human dental plaques and are known to induce periodontal inflammation [44, 45]. In addition, *P. piscolens* was first isolated from the human oral cavity and is associated with oral dysbiosis [46]. The outcome of CRC treatment thus could be influenced by the microbial homeostasis throughout the oral cavity and gastrointestinal tract. *A. dispar* and *A. senegalensis*, which are taxonomically assigned to *Bacteroides* sp. according to the latest SILVA 138 database, are additional biomarkers for dismal outcomes. *Alistipes* is a relatively new bacterial genus that branches off from *Bacteroides* and is associated with many diseases in both protective and pathogenic ways [47]. In CRC, *Alistipes* is increased in the carcinoma tissues compared to that in the healthy controls and advanced adenomas [48].

We suggested microbiota-derived thiamine (vitamin B1) as a beneficial micronutrient for CRC through computational inference (*P*_PWY-6897_= 0.0087, *P*_THISYN-PWY_ = 0.087; Fig. 4D and Figure S5A). Low intake of thiamine is associated with increased CRC risk, and high doses of thiamine suppress cancer cell proliferation in vitro [49, 50]. The dietary thiamine absorption mainly occurs in the small intestine; therefore, the colonic concentration of thiamine might be affected by the gut microbiota that heavily colonizes the large intestine. A microbial pathway that degrades histidine was suggested to underlie the poor prognosis of CRC (Fig. 4E). The key enzymes of the histidine catabolism pathway, such as histidase and urocanase, are found in bacteria and humans with relatively high amino acid identity (35-40%), implying a conserved function across kingdoms [51]. Patients with metastatic CRC have low circulating histidine [52]; patients expressing high levels of histidine catabolic enzymes in CRC tissues had a poor survival [53]. Despite the supporting evidence, the involvement of the inferred microbial pathways and molecules in modulating the tumor microenvironment must be understood with caution and requires experimental validation.

We combined the prognostic biomarkers to make the best MHS; therefore, the addition of biomarkers steadily improved the robustness of MHS (Fig. 2G), because the low prevalence of the indicative bacteria could induce a bias toward certain datasets. To make the index more user-friendly, we applied an absence/presence scale for the detrimental biomarkers. This does not necessitate abundance measurements; it allows the qualitative detection of the target species through the easy-to-perform PCR. The M5 can be simplified by converting the abundance scale of *Prevotella* into the absence/presence scale. The modified M5 still had a strong prediction power (data not shown). Compared to liquid or tissue biopsies, feces can be collected by the patients themselves at home without technical hurdles. This should be a compelling advantage for longitudinal monitoring of postoperative patients.

## Conclusions

Despite some limitations such as single institutional sampling and lack of mechanistic validation on inferred microbial pathways, we showed the beneficial impact of *Prevotella*, a representative bacterium of the human enterotype, for the first time and generated a simple but robust microbial hazard score combining *F. nucleatum* and our novel prognostic species for CRC prognosis. These results would be useful for predicting medical outcomes after surgical treatment of CRC and for managing postoperative patients.

## Supplementary Information

**Additional file 1: Table S1.** Clinical data of patients with primary CRC.

**Additional file 2: Figure S1.** Indicator profiles by enterotype.

**Additional file 3: Figure S2.** Prognosis by microbial variables.

**Additional file 4: Figure S3.** Characterization of ASVs assigned to *Bacteroides* sp.

**Additional file 5: Figure S4.** Progression-free survival by various host factors.

**Additional file 6: Figure S5.** Progression-free survival by the differentially enriched microbial pathways.

## Data Availability

The 16S rRNA gene sequencing data generated in this study have been deposited in the National Center for Biotechnology Information (NCBI) Sequence Read Archive under the accession number PRJNA859426.

## Abbreviations

ASA: American Society of Anesthesiologists
BMI: Body mass index
C-index: Concordance index
CoxPH: Cox proportional hazard regression model
CRC: Colorectal cancer
FDR: False discovery rate
HR: Hazard ratio
NLR: Neutrophil-to-lymphocyte ratio
OS: Overall survival
PCoA: Principal coordinate analysis
PERMANOVA: Permutational multivariate analysis of variance
PFS: Progression-free survival
TRFA: Taxonomic relative functional abundance
