# Supplementary figures and images for: Enterotypical Prevotella and three novel bacterial biomarkers in preoperative stool predict the clinical outcome of colorectal cancer
Source: Microbiome. 2022 Nov 28;10:203. doi: 10.1186/s40168-022-01388-8 (PMC9703702; doi:10.1186/s40168-022-01388-8)

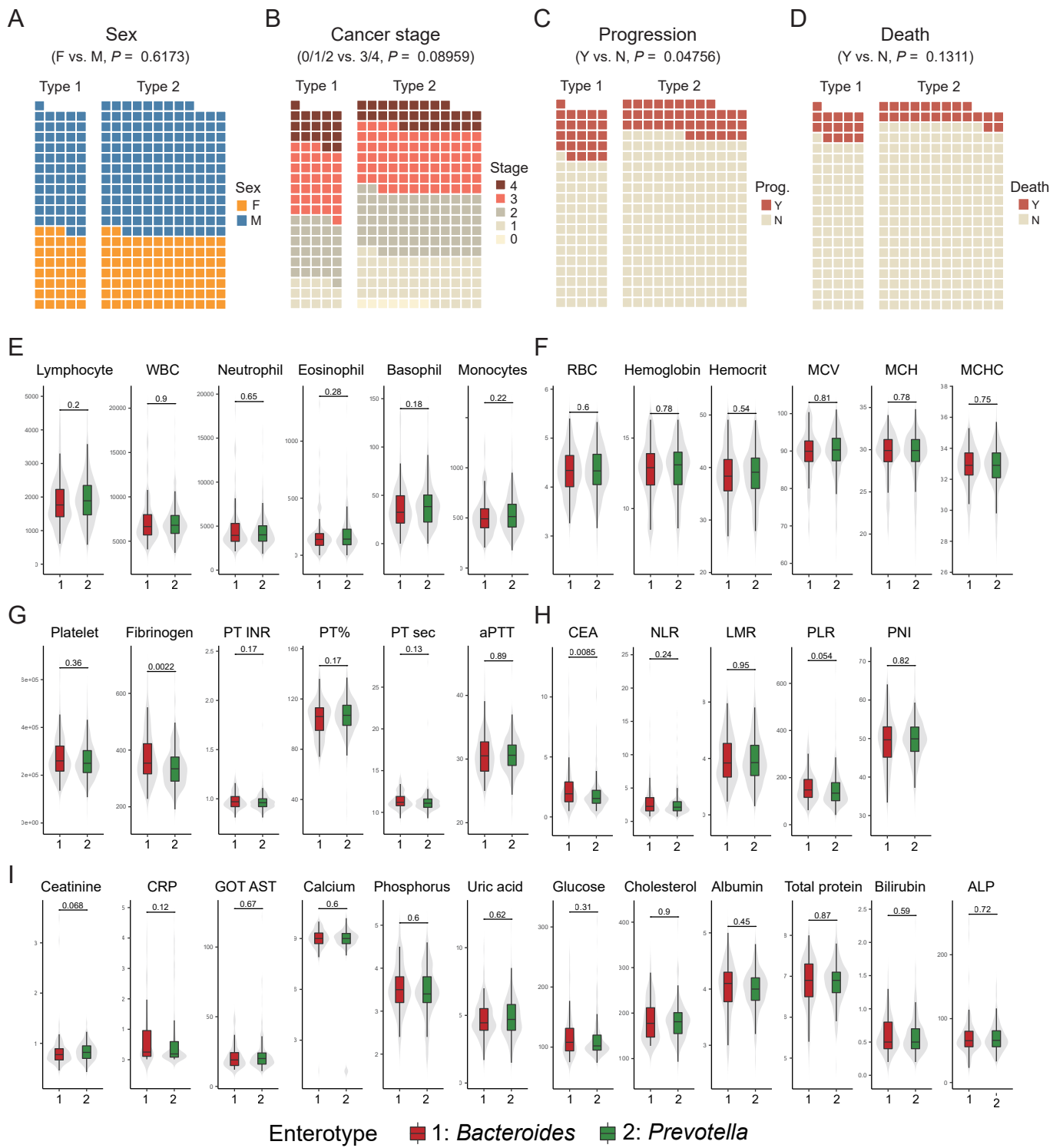

Supplement: Supplementary file 3 — Additional file 2: Figure S1. Indicator profiles by enterotype. [file 40168_2022_1388_MOESM2_ESM.pdf]

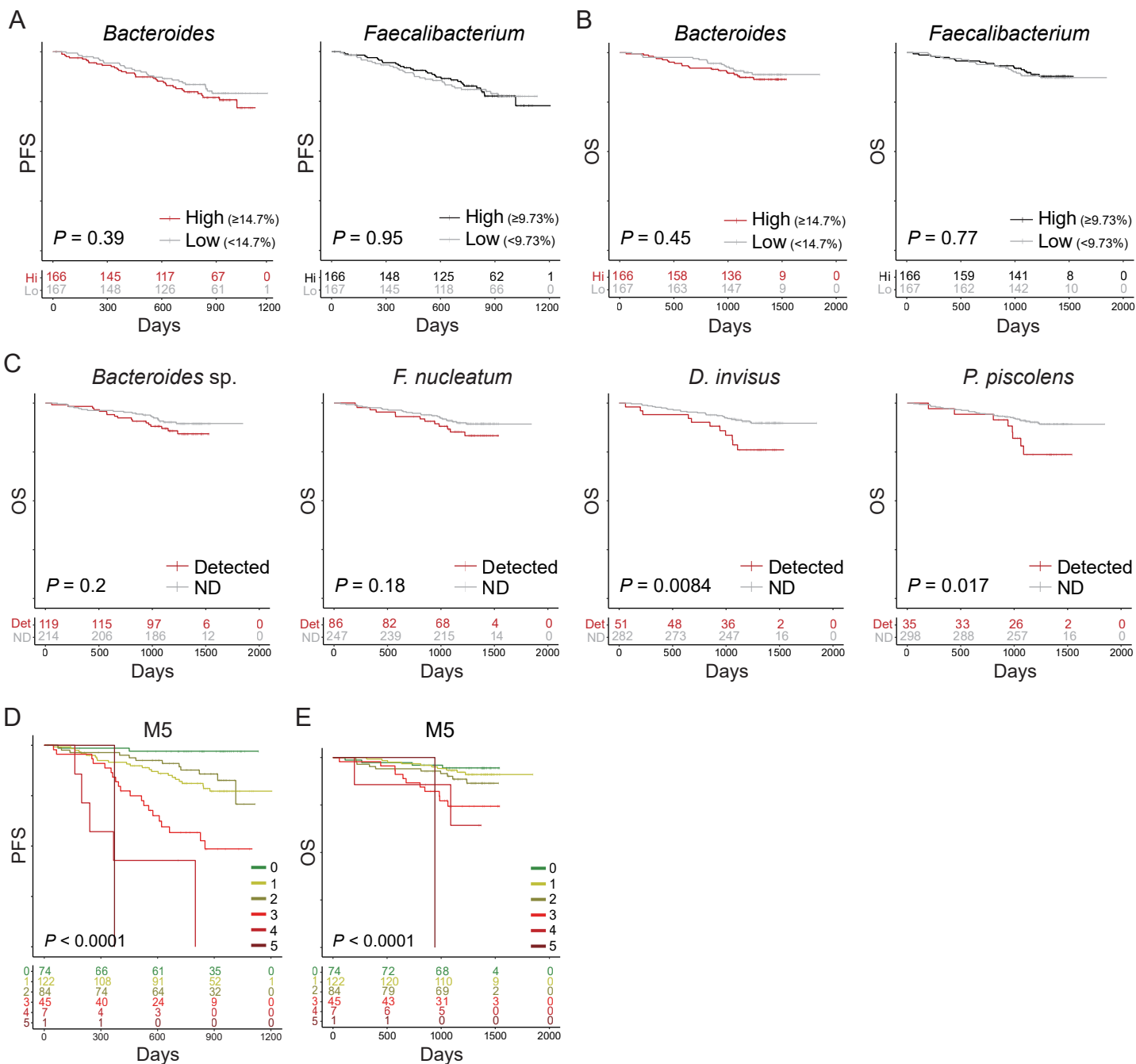

Supplement: Supplementary file 4 — Additional file 3: Figure S2. Prognosis by microbial variables. [file 40168_2022_1388_MOESM3_ESM.pdf]

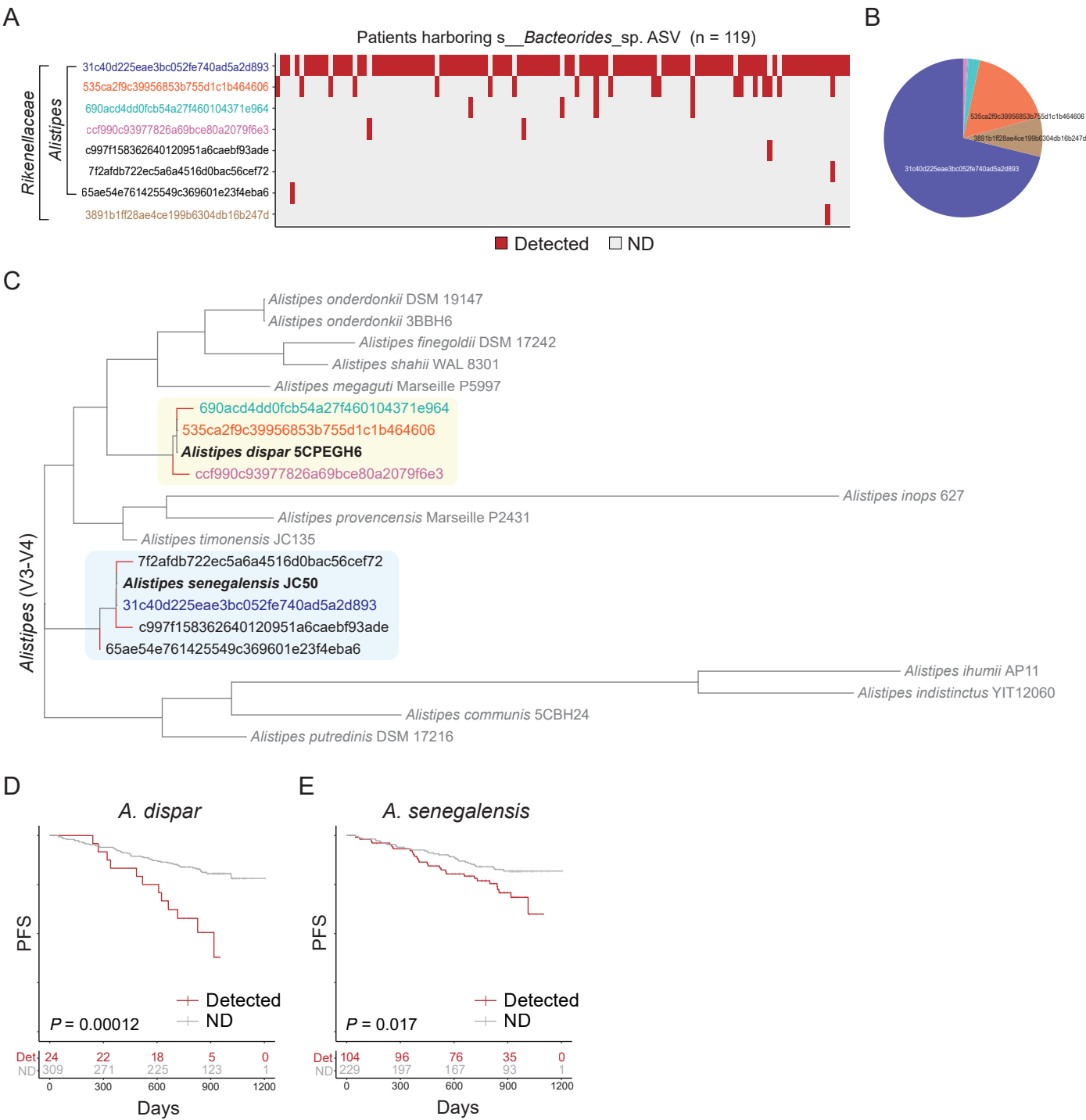

Supplement: Supplementary file 5 — Additional file 4: Figure S3. Characterization of ASVs assigned to Bacteroides sp. [file 40168_2022_1388_MOESM4_ESM.pdf]

A

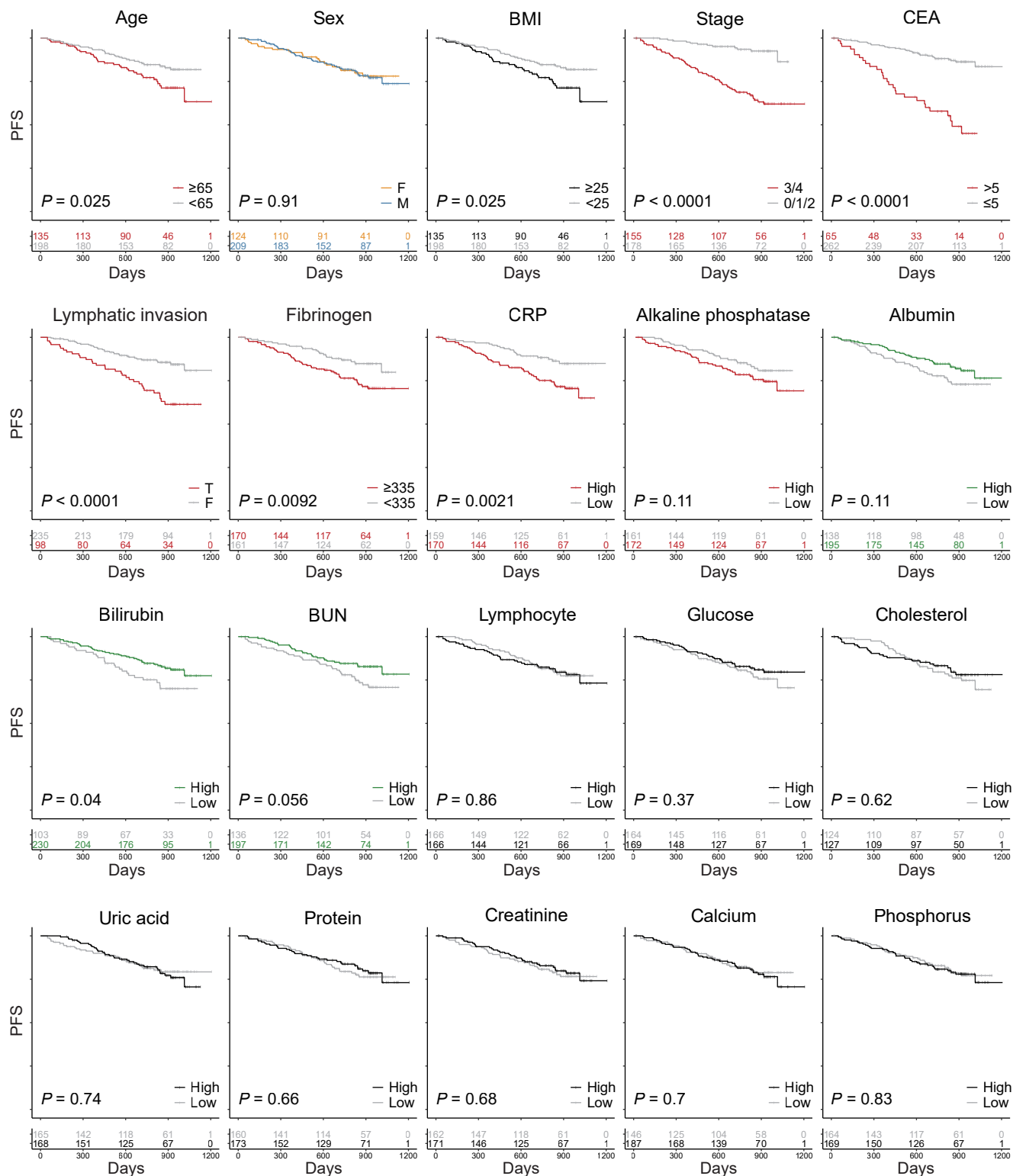

B

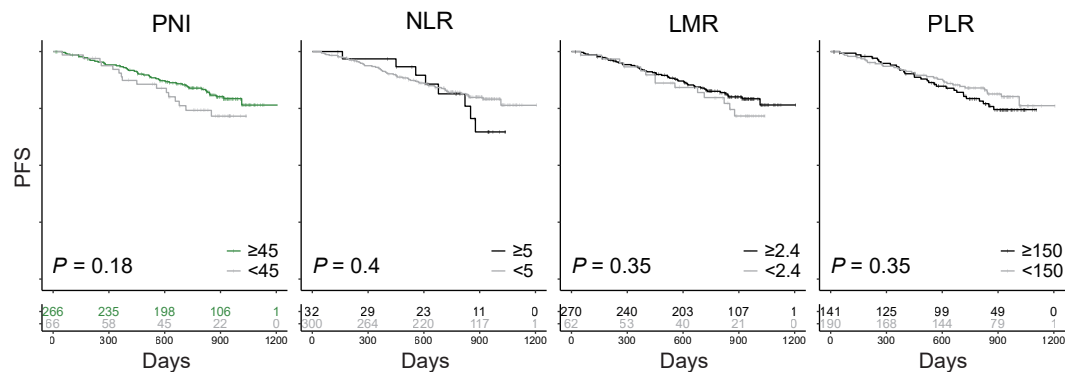

Supplement: Supplementary file 6 — Additional file 5: Figure S4. Progression-free survival by various host factors. [file 40168_2022_1388_MOESM5_ESM.pdf]

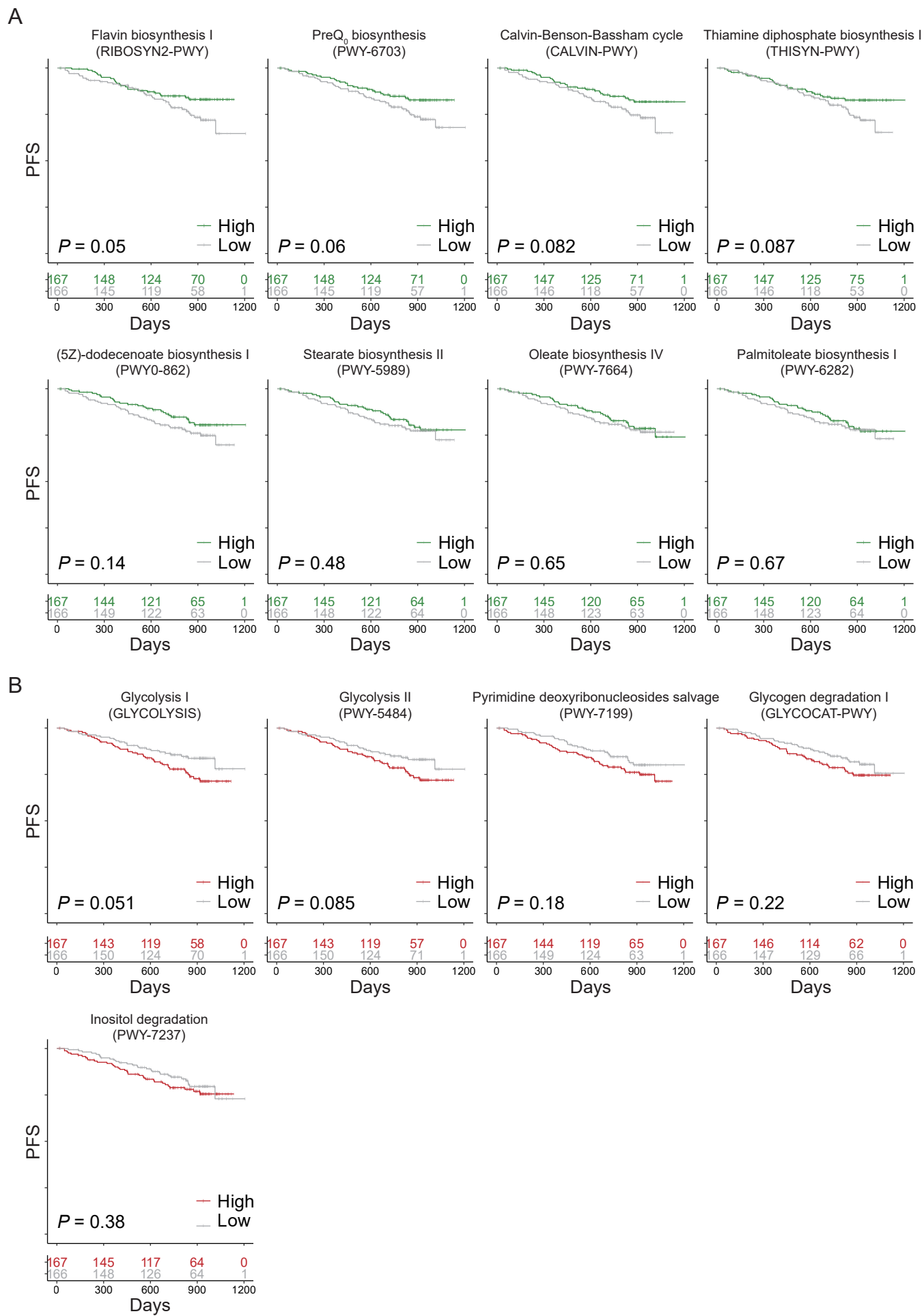

Supplement: Supplementary file 7 — Additional file 6: Figure S5. Progression-free survival by the differentially enriched microbial pathways. [file 40168_2022_1388_MOESM6_ESM.pdf]
